# Major Differences in Diet across Three Linguistic Regions of Switzerland: Results from the First National Nutrition Survey *menuCH*

**DOI:** 10.3390/nu9111163

**Published:** 2017-10-25

**Authors:** Angeline Chatelan, Sigrid Beer-Borst, Alex Randriamiharisoa, Jerome Pasquier, Juan Manual Blanco, Stefan Siegenthaler, Fred Paccaud, Nadia Slimani, Genevieve Nicolas, Esther Camenzind-Frey, Christine Anne Zuberbuehler, Murielle Bochud

**Affiliations:** 1 Institute of Social and Preventive Medicine (IUMSP), Lausanne University Hospital (CHUV), Route de la Corniche 10, 1010 Lausanne, Switzerland; angeline.chatelan@chuv.ch (A.C.); alex.randriamiharisoa@chuv.ch (A.R.); jerome.pasquier@chuv.ch (J.P.); juan-manuel.blanco@chuv.ch (J.M.B.); fred.paccaud@chuv.ch (F.P.); 2 Institute of Social and Preventive Medicine (ISPM), University of Bern, Finkenhubelweg 11, 3012 Bern, Switzerland; sigrid.beer@ispm.unibe.ch; 3 Health Division, Bern University of Applied Sciences, Stadtbachstrasse 64, 3012 Bern, Switzerland; stsiegenthaler@gmail.com; 4 International Agency for Research on Cancer (IARC), World Health Organization, 150 Cours Albert Thomas, 69372 Lyon, France; slimanin@iarc.fr (N.S.); nicolasg@iarc.fr (G.N.); 5 Risk Assessment Division, Scientific Evaluation Sector, Federal Food Safety and Veterinary Office (FSVO), Schwarzenburgstrasse 155, 3003 Bern, Switzerland; esther.camenzind-frey@blv.admin.ch (E.C.-F.); christine.zuberbuehler@blv.admin.ch (C.A.Z.)

**Keywords:** national nutrition survey, food consumption, 24-h dietary recall, GloboDiet^®^/EPIC-Soft^®^, food-based dietary guidelines, Swiss adults

## Abstract

Switzerland is a multilingual country located between Germany, France and Italy, which differ by dietary habits and related outcomes. We explored differences in food consumption as well as compliance to the Swiss food-based dietary guidelines (FBDG) across the German-, French-, and Italian-speaking regions. The 2014–2015 nationwide cross-sectional survey was conducted among a stratified random sample of 2057 adults aged 18 to 75 years. Trained dietitians assessed food consumption via two non-consecutive 24-h dietary recalls using the international validated software GloboDiet^®^. Recorded foods and beverages were classified into six groups and 31 subgroups relevant for assessing compliance to the FBDG (Swiss Food Pyramid). Usual daily intake distributions were modelled and weighted for sampling design, non-response, weekdays and season. Participation rate was 38%. Significant differences across regions were observed in 18 of 31 food subgroups (*p* ≤ 0.01). Weighted mean daily intakes in the German-, French- and Italian-speaking regions were, respectively, 245 g, 155 g, 140 g for soft drinks, 273 g, 214 g, 135 g for coffee, 127 g, 72 g, 109 g for milk, 32 g, 45 g, 43 g for red meat, 18 g, 29 g, 34 g for fish/seafood, 8.1 g, 6.4 g, 3.7 g for butter, and 206 g, 214 g, 168 g for vegetables. The seven FBDGs were followed by <1% of the population. Four in 10 participants met ≥3 FBDG. Eighteen percent of participants ate ≥5 portions of fruit and vegetables a day, without regional differences. Food consumption substantially differed across the three linguistic regions of Switzerland. Adherence to FBDG was uniformly low. This highlights the potential influence of culture on diet. Nutritional education along with public health interventions are needed and may be most efficient if regionally targeted.

## 1. Introduction

Switzerland is centrally located in Europe at the crossroads between Germany, France and Italy. Despite a small size, the country has three main linguistic regions: German (63%, in the north, east and center), French (23%, in the west) and Italian (8%, in the south) [1]. In all three regions, the population has very high life expectancy [2,3], high income [2,4], universal health coverage and one of the lowest prevalence of obesity in western countries [3,5]. Chammartin [6] and Faeh [7] et al. observed, however, significant regional variations in mortality rates for chronic diseases known to be influenced by diet, such as coronary heart disease, stroke, type 2 diabetes, gastric and liver cancer.

Diet quality, independently of energy or macronutrient intake, is a major modifiable determinant of most chronic diseases (e.g., cardio-metabolic diseases and cancers) [8,9,10]. Until 2015, Switzerland had no national survey to assess food consumption and diet quality, such as adherence to food-based dietary guidelines (FBDG). Until then, scientists and policy makers relied on national agricultural statistics [11,12], regional epidemiologic studies [13,14,15] and assessment of single nutrition-related items in the Swiss Health Survey [16,17]. These data have limitations such as high aggregation or do not allow comparison between the linguistic regions. The first national nutrition survey *menuCH* was conducted in 2014–2015 to fill this gap.

The European Prospective Investigation into Cancer and Nutrition (EPIC) study has shown large differences in diet between and within countries [18,19]. For example, diets in Northern European countries, including Germany, are richer in animal and processed foods compared to the ones in Italy and Greece [18]. In a multilingual country such as Belgium, the national nutrition survey also showed substantial differences in food consumption between people from the French- and the Dutch-speaking regions [20]. Due to Switzerland’s multilingual situation and geographical location in-between three countries with differing dietary habits, we expected language-regional differences in food consumption. Using data from the first national nutrition survey *menuCH*, we investigated the differences in food consumption, as well as adherence to national FBDG, across the German-, French-, and Italian-speaking regions of Switzerland.

## 2. Materials and Methods

We followed the STROBE-nut recommendations for reporting [21].

### 2.1. Study Design and Sampling

The population-based cross-sectional survey was conducted in ten study centres among non-institutionalised residents of Switzerland aged 18 to 75 years from January 2014 to February 2015. The Federal Statistical Office drew a stratified random sample from the national sampling frame for person and household surveys [22]. The sample was intended to be representative for the following 35 strata (7 × 5): (1) the seven major areas of Switzerland (Lake Geneva, Midlands, Northwest, Zurich, East, Central, and South), covering the three main linguistic regions (German, French and Italian) and considering 12 cantons/states; and (2) five predefined age categories. Recruitment of participants followed a previously tested procedure [23]. In short, 13,606 individuals were invited to participate via a post-mailed letter with response card. People with known phone numbers were called by specifically trained recruiters (up to seven call attempts) to schedule an interview in a study centre. Recruiters collected information about the reasons of refusals.

### 2.2. Dietary Assessment

Fifteen field dietitians had six weeks of intensive training and regular retraining during data collection. They assessed food consumption through two non-consecutive 24 h dietary recalls (24HDR), as described elsewhere [23]. In brief, the first face-to-face and the second phone 24HDR (two to six weeks later) were distributed across all weekdays and seasons. The 24HDR were multiple-pass and automated using the software GloboDiet^®^ (GD, formerly EPIC-Soft^®^, version CH-2016.4.10, International Agency for Research on Cancer (IARC), Lyon, France) [24,25], adapted to the Swiss food market (GD trilingual databases dated 12.12.2016, IARC, Lyon, France and Federal Food Safety and Veterinary Office, Bern, Switzerland). GD displayed food group specific descriptors allowing highly standardised description of foods and recipes, such as cooking and preservation methods, sugar and fat contents. To support survey participants in quantifying consumed amounts, a book with 119 series of six graduated portion-size pictures [26] and a set of about 60 actual household measures were used. A newly developed matching tool FoodCASE (Premotec GmbH, Winterthur, Switzerland) allowed linkage between foods, recipes and ingredients from GD with the most appropriate item from the Swiss Food Composition Database [27]. In this paper, energy and macronutrients were assessed mainly for the estimation and interpretation of misreporting.

### 2.3. Food Grouping and Comparisons to FBDG

The national FBDG, which is the 2011 six-stage Swiss Food Pyramid [28], was the reference for food grouping. Every stage represents a main food group with several subgroups: non-caloric beverages (three subgroups), fruit and vegetables (three subgroups), cereal products and potatoes (four subgroups), protein-based products (nine subgroups), added fats and nuts (four subgroups), sweets, salty snacks and alcohol (eight subgroups) (Table S1). Two registered dietitians independently classified recorded foods and beverages into these six main food groups and 31 subgroups according to their nutritional profile. This grouping allowed overall comparison between the guidelines for each stage of the Pyramid [28] and the actual food consumption at a population level. Finally, we selected seven FBDG with quantitative cut-offs for daily consumption to assess the proportions of people meeting these across the three linguistic regions. The selected FBDG were about the consumption of non-caloric beverages, fruit/vegetables, dairy products, meat, vegetable oil, nuts/seeds, and alcohol.

### 2.4. Anthropometry and Other Parameters

Body weight, height and waist circumferences were measured following international standard protocols [29] as described elsewhere [23,30]. Self-reported values were used for pregnant and lactating women or when measurements were impossible (*n* = 34). Participants’ education and physical activity level were assessed by standardised questionnaire. The latter was assessed using the short-form International Physical Activity Questionnaire (IPAQ) [31,32] and categorised into three levels (PAL) following IPAQ classifications: low, moderate and high [33].

### 2.5. Quality Controls

Three survey coordinators attended 88 (2%) interviews evenly distributed over the survey period, and assessed compliance to 49 survey specific standard operating procedures. Dietitians were all rated between 2.78 and 2.99 (*1* = *inadequate; 3* = *good practice*). A senior registered dietitian cleaned GD data according to the guidelines prescribed by IARC [34,35,36]. Data were also screened for inconsistencies (e.g., extreme energy intakes) applying all IARC’s recommended criteria [34]. Following European guidelines [37], the ratio of reported Energy Intake (mean of the two 24HDR) to Basal Metabolic Rate (EI:BMR) was calculated at a population level. To compute the percentage of misreporters, BMR per participant was estimated using Schofield equations [38]. We used weight, height, age and sex, and applied the age-specific expected PAL-values and Goldberg cut-offs [39,40]. When IPAQ data were incomplete (*n* = 524), participants were considered as moderately active (PAL = 1.6). Under- and over-reporters were included in the analyses. More details about quality controls can be found in a separate paper [23].

### 2.6. Weighting

All survey results were weighted for age, sex, marital status, major area based on home address, nationality and household size to take account of the sampling design and non-response. The 2014 sampling frame was used as the reference population of the 12 selected cantons. We could not weight results for socio-economic status (e.g., education) because we had no data available for non-participants. For analyses on food subgroup and macronutrient intakes, we also corrected for the uneven distribution of 24HDR over the year (Figure S1). Weighting variables included seasonality (four seasons, date between both 24HDR) and days of the week (two week days (Monday–Thursday), two weekend days (Friday–Sunday), or one week day and one weekend day). A detailed documentation about the weighting strategy is available in the open survey data repository.

### 2.7. Statistical Analysis

Mean daily food intakes by food subgroups and mean daily macronutrient intakes were computed out of the two 24HDR. Additionally, *usual* intake distributions adjusting for within individual day-to-day variation were modelled with the Statistical Program to Assess Dietary Exposure (SPADE, version 3_1, option backtrsn = 2) [41,42] implemented in R [43]. The modelling option for *episodically* consumed food subgroups (all subgroups, except water and vegetables) and macronutrients (only for alcohol) was applied when more than 4% of participants reported zero intakes on both recorded days. In the absence of food frequency data, usual intakes were modelled only from 24HDR. Standard errors of the means were derived from weighted bootstrap samples using age as covariate (four categories). Sensitivity analyses were performed applying a second model to derive usual intakes, i.e., Multiple Source Method (MSM) [44]). We conducted survey-weighted logistic regressions to assess differences in adherence to FBDG across linguistic regions. Except for usual intake modelling, analyses were performed using STATA 14 (Stata Corporation, College Station, TX, USA). The statistical significance of differences across linguistic regions was assessed with a Wald test, applying correction for multiple testing (*p ≤* 0.001). We used ArcMap™ 10.4 (Esri, Redlands, CA, USA) to create maps describing consumption at a canton/state level and applied natural breaks (Jenks) for class definition.

### 2.8. Ethics

This survey was conducted according to the guidelines laid down in the Declaration of Helsinki and all procedures were approved by the corresponding regional ethics committees (lead committee in Lausanne, Protocol 26/13, approved on 12 February 2013). Written informed consent was obtained from all participants. The survey is registered (International Standard Randomised Controlled Trial Number (ISRCTN): ISRCTN16778734).

## 3. Results

### 3.1. Sample Characteristics

Net participation rate was 38.0% (Figure S2). Out of 3410 refusals, 1942 (57%) gave a reason for refusal. The main reasons were lack of time (56%), no interest (28%), and difficulty to reach the study centre (6%). Participants and non-participants had similar age and marital status, but participants were more frequently women and Swiss nationals (Table 1). Among the 2086 survey participants, 2057 (99%) had two complete 24HDR and were included in the analyses. The latter represent 4.6 million residents. Table 2 shows their key characteristics. The prevalence of overweight and obesity was higher in men (41% and 13%, respectively) than in women (20% and 11%). One third of participants were at increased, or substantially increased risk, of metabolic complications, based on the waist circumference cut-off points from the World Health Organization [45]. The mean EI:BMR ratio (±SD) was 1.43 (±0.44) and similar in both sexes. We observed 17% of under-reporters overall, with a higher proportion in the Italian-speaking region.

### 3.2. Food Group and Macronutrient Intakes

**Figure 1:**
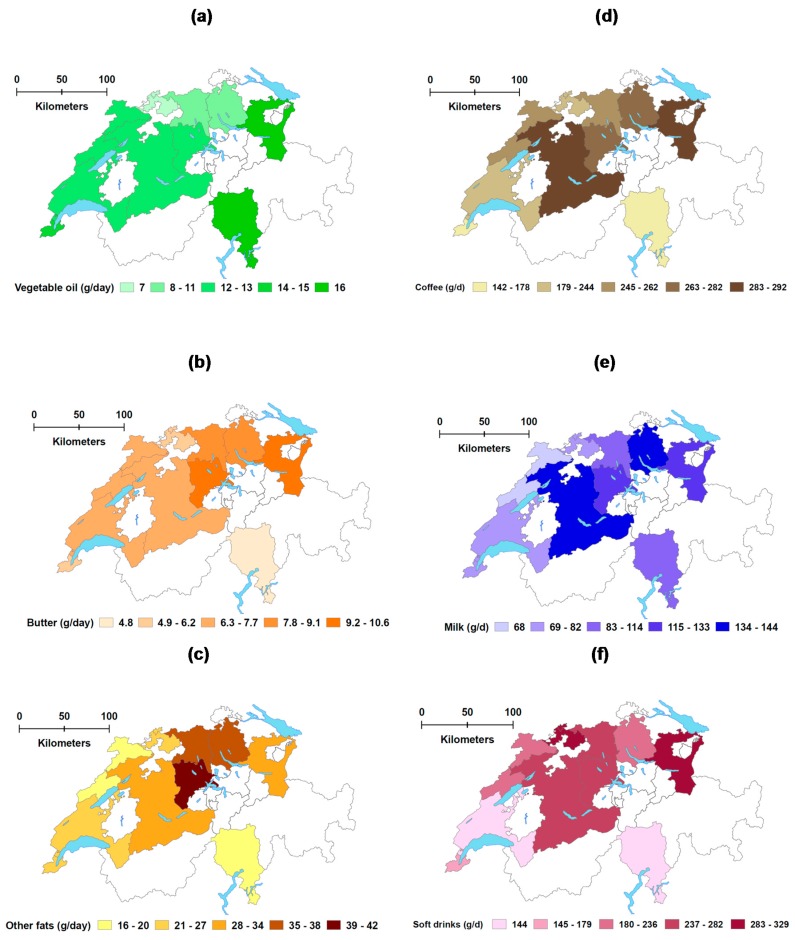
Daily consumption (weighted mean) of vegetable oil (**a**); butter (**b**); other fats (i.e., cream, fatty sauces and other fats) (**c**); coffee (**d**); milk (**e**); and soft drinks (**f**) for 12 cantons/states.

Large regional differences in food intake were observed in all six Food Pyramid stages, and in 18 out of 31 subgroups (Table 3, *p ≤* 0.01). Coffee consumption was higher in the German- than the French- and Italian-speaking regions (*p* < 0.001). Residents in the French-speaking region consumed on average 46 g more vegetables per day than in the Italian one (*p* < 0.001). Milk intake was the highest in the German-speaking (127 g/day) and the lowest in the French-speaking regions (72 g/day). Processed meat (42 g/day) was the most consumed food group among meat- and fish-based products (127 g/day) in the Swiss population. Consumption of fish, red meat and other unprocessed meat (i.e., mainly poultry) was higher in both, the French- and Italian-speaking regions, than in the German one. Consumption of added fats substantially differed across linguistic regions. The largest quantities of butter and cream/fatty sauces were consumed in the German-speaking region (8.1 g/day and 36 g/day, respectively, *p* < 0.001), whereas vegetable oil was more abundant in the Italian-speaking region (*p* = 0.01). Soft drinks consumption (including fruit lemonades and sugar-free soft drinks) was much higher in the German- (245 g/day) than in the French- (155 g/day) or Italian-speaking regions (140 g/day, *p* < 0.001). Figure 1 details the consumption at a cantonal/state level of added fats (stage 5 of the Pyramid) (a–c) and coffee, milk and soft-drinks (d–f), three food subgroups with large regional differences. Sensitivity analyses found similar regional differences when usual intakes were modelled by MSM (*data not shown*).

Daily energy intake was 2185 kcal, when both genders were considered together: i.e., 2538 and 1899 kcal for men and women, respectively (Table S2). Proteins (83 g/day), carbohydrates (230 g/day), fat (90 g/day), and alcohol (13 g/day) contributed 15%, 42%, 37%, and 4% of total energy intake, respectively (*remaining 2% for fibres*). Although protein sources differed across regions (stage 4 of the Pyramid) at a food level, total protein intake was similar (82–83 g/day). Usual food and macronutrient intakes stratified by age groups are presented in Table S3.

### 3.3. Adherence to FBDG

**Figure 2:**
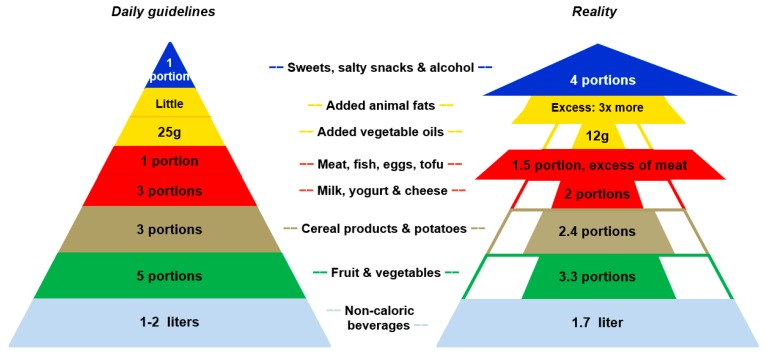
The daily national food-based dietary guidelines (i.e., Swiss Food Pyramid) (**left**) compared to the actual food consumption for the six stages at a population level (**right**).

Figure 2 provides an overview of the national FBDG (i.e., Swiss Food Pyramid) (left) and the actual consumption for the six stages (right). At a population level, only the non-caloric beverages were consumed in accordance with the guideline. Table 4 presents the proportion of participants who followed the FBDG. Less than 1% of the total population followed all seven selected FBDG and 41% at least three of them, without regional differences. More than three quarters of people fulfilled the guidelines to consume at least 1 L/day of non-caloric beverages. Only 18% consumed at least five portions of fruit and vegetables per day, without regional differences (*p* = 0.79 to 0.92). Nevertheless, in the German-speaking region, more residents (29%) followed the fruit specific guideline compared to the Italian-speaking region (20%, *p* = 0.013). One out of five Swiss residents reported eating daily three portions of dairy products and less than 35 g of cooked/prepared meat, which corresponds to 2–3 times a week a portion of 110 g of any type of raw meat. Population in the Italian-speaking region followed twice as much the guideline on vegetable oil than their German-speaking counterparts (*p* < 0.001). In the French-speaking region, the guideline for alcoholic beverages (72%) was less respected than in the German- (78%, *p* = 0.029) or Italian-speaking regions (79%, *p* = 0.094).

## 4. Discussion

The intake of 18 of 31 FBDG-related food subgroups significantly differed across the three main linguistic regions of Switzerland, suggesting regional specificities in the ‘Swiss diet’. This is the first time such large differences could be observed in populations with similar ethnic (i.e., mostly Caucasian), economic [4] and educational backgrounds living only 100–400 km apart. Regional differences in the amount of foods consumed were especially marked for coffee, vegetables, dairy products, red meat, fish/seafood, added fats, and soft drinks. This highlights the potential influence of cultural background on diet, although we cannot exclude socio-economic differences across linguistic regions may play a role. Despite large regional differences in food subgroup intake, we found no major regional differences in compliance to the national FBDG, which was uniformly low.

### 4.1. Regional Differences in Food Consumption

Differences in food consumption within a multilingual country were previously documented in Canada and Belgium, but to a lesser extent. The 2004 Canadian Community Health Survey did not find significant differences in food and nutrient intakes between English- and French-speaking regions [46], even though Quebecers ate less fruit and vegetables than the English-speakers [46,47]. In Belgium, noticeable differences in the French- and Dutch-speaking regions were seen for fruit (86 g vs. 121 g, respectively), fish/seafood (16 g vs. 24 g) and beer (53 g vs. 113 g) but not for water, vegetables, and soft drinks [20]. Similarly to our study, milk was also less consumed by the people living in the French-speaking region, closer to France, than those residing in the Dutch-speaking region (140 g vs. 171 g, respectively, *milk and yogurt together*) [20].

In spite of some food market globalisation, the Swiss residents may be influenced by dietary habits from the larger bordering countries—Germany, France and Italy. Linseisen et al. [48] reported similar findings for added fats in the 1995–1998 EPIC study. Using the same dietary assessment tool, they found that the Italians consumed about seven times as much vegetable oil as the Germans while as many times less butter [48]. Preferences for red meat in France and Italy, and for processed meat such as sausages in Germany, have also been observed in the EPIC study [49]. Milk consumption is another good example showing how the multilingual and cultural setting of Switzerland is in line with differences observed between bordering countries. National nutrition surveys have shown a daily milk intake among adults of 115 g in Germany [50], 75 g in France [51] and 103 g in Italy [52], while our survey showed respectively 127 g, 72 g, 109 g in the German-, French- and Italian-speaking regions. Being poorer in milk, and richer in vegetable oil, fish, and poultry, diets in the French and Italian-speaking regions have some closer characteristics to the Mediterranean diet. This may partly explain the lower cardiovascular mortality in these regions of Switzerland [6,53]. Higher alcohol consumption in the French-speaking region was previously documented by Faeh et al. [7], who also reported less circulatory mortality, but more alcohol consumption related deaths (e.g., due to upper aerodigestive tract and liver cancers) in this region, compared to the German-speaking one.

Despite sizeable differences in food consumption no major regional differences in BMI or waist circumference were observed. Indeed, these two indicators were relatively similar across the regions considering that the French-speaking population was slightly younger. This observation is in line with the 2012 Swiss Health Survey [3,17], where obesity prevalence across linguistic regions was shown to be similar (close to 11%).

### 4.2. Adherence to FBDG

FBDG deliver messages to the population about healthy eating, mainly in the context of primary prevention of diet-related chronic diseases. There is a wide consensus from observational and experimental studies that fruits, vegetables, nuts, vegetable oils, whole grains, and fish are protective against most cardio-metabolic diseases and cancers, whereas red and processed meats as well as foods rich in refined grains, added sugars, and salt should be limited [8,9,10,54]. Imamura et al. [55] reported overall low diet quality in adult populations from high-income countries using a systematic assessment across countries. Other studies in Western Europe [56,57], and in two Swiss French-speaking cities using a food frequency questionnaire [13,14], have found overall low compliance to the national FBDG. Therefore, low compliance to the Swiss FBDG was expected. Our survey showed that less than one out of five Swiss residents met the fruit and vegetable guidelines. Mean daily consumption of fruit and vegetables (excluding juices) was higher in Switzerland (203 and 163 g/day, respectively) than in France (144 and 131 g/day) [51]. It was however lower than in Germany (236 and 254 g/day) [50] and Italy (205 and 223 g/day) [52]. The tendency to consume more fruit in Germany compared to France and Italy was also found in our survey, which showed higher percentage of people meeting the guidelines of two portions a day in the German-speaking than in both Latin parts of Switzerland.

Comparison of compliance rate to FBDG may be difficult since each country has different food classification and cut-offs. For nutrients, such as energy, comparisons are simpler. In *menuCH*, mean daily energy intakes for men and women (i.e., 2538 and 1899 kcal) were close to recommendations for moderately active adults [58]. These findings were also very similar to those found in the 2005–2007 German food consumption survey (2571 and 1915 kcal, respectively) [59] as well as the 2014–2015 French (2462 and 1788 kcal) [51] and the 2005–2006 Italian surveys (2390 and 1939 kcal) [60]. This emphasises the value of the Swiss data and the relevance of between-country comparisons.

### 4.3. Strengths

The survey participants were recruited based on stratified random population-based sampling. Interviewers were extensively trained and retrained over the year of data collection to maximise standardisation and quality of 24HDR in all study centres. The use of the GloboDiet^®^ software has eased data comparison between the three linguistic regions and other high-income countries since it is the reference tool in European food consumption surveys and it is very similar the Automated Multiple-Pass Method used in North-America. Dropouts for the second 24HDR were rare (1%). The implemented weighting strategy allowed, to some extent, the correction for non-response and uneven distribution of 24HDR over season and weekdays. Finally, differences in food consumption across the linguistic regions were assessed on *usual* intake distributions modelled by both SPADE and MSM (for sensitivity analyses). Both modelling methods provided similar findings, as previously tested by others [41,61].

### 4.4. Limitations

The survey included only the most populous cantons/states of Switzerland (Figure S3) in this first nationwide initiative for logistic and financial reasons. Participation rate was low but in the range of other food consumption surveys in Europe [50,52]. Twenty-nine percent of the sample could not be reached by recruiters due to lack of available phone numbers. Although survey participants resembled non-participants in terms of socio-demographic characteristics, we cannot exclude participation bias. Participants may have been more nutrition conscious than non-participants. Nevertheless, the prevalence of overweight (31%) and obesity (13%) in our participants was comparable to the one reported in the phone-administrated Swiss Health Survey, whose participation rate was higher [17]. Energy under-reporting was detected in 19% of men and 15% of women, which is lower or comparable with rates found in European studies applying similar calculations [50,62]. This may reflect true day-to-day variations. In *menuCH*, 25% of underreporting cases could be explained by the fact they were on a diet at the time of 24HDR. Social desirability and recall bias are other well-known sources of underreporting in 24HDR [63]. Lastly, we could not estimate the probability of consumption in the modelling of *usual* intakes because no appropriate national food frequency/propensity questionnaire was available and validated at time of the survey. For the same reason, we focused only on the amount of foods consumed per day and not the consumption frequency.

## 5. Conclusions

The first Swiss nutrition survey showed significant differences in food consumption across the three main linguistic regions, in particular for beverages (i.e., non-caloric or soft drinks), protein-based products (e.g., fresh dairy, red meat and fish) and added fats. This emphasises the potential influence of culture on diet in populations with similar ethnic and economic backgrounds in a high-income country, and may partly contribute to previously reported regional variations in causes of death in Switzerland [6,7]. Adherence to national FBDG was low in the population, and similar across the three regions. This may reflect lack of awareness or willingness to follow the dietary guidelines, limited practicability of current guidelines, and/or lack of access to healthy foods. Efforts should be increased to continuously raise awareness of the national FBDG and make healthier foods the easiest options in order to help the whole population adhere to the dietary recommendations. Findings from this survey will help the Swiss government elaborate targeted actions for implementation of the 2017–2024 Nutrition Strategy [64]. Finally, education messages along with public health interventions may be most efficient if regionally targeted.

## Figures and Tables

**Table 1:** Characteristics of participants vs. non-participants, Swiss Nutrition Survey, 2014–2015.

Characteristics	Non-Participants	Participants	*p*-Value ^1^
Total (*n*, %)	11,520 (84.7)	2086 (15.3)	
Sex (*n*, %)			
Men	5757 (50.0)	946 (45.3)	<0.001
Women	5763 (50.0)	1140 (54.7)	
Age			
(year, mean ± SD)	46.1 (±15.4)	46.8 (±15.8)	0.096
Marital status (*n*, %)			
Single	3864 (33.5)	695 (33.3)	0.114
Married	6118 (53.1)	1147 (55.0)	
Widowed	332 (2.9)	43 (2.1)	
Divorced	1177 (10.2)	194 (9.3)	
Others	29 (0.3)	7 (0.3)	
Number of household members (*n*, %)			
1 person	2085 (18.1)	309 (14.8)	0.003
2 people	3712 (32.2)	712 (34.1)	
3 people	2154 (18.7)	381 (18.3)	
4 people	2209 (19.2)	440 (21.1)	
5 or more	1360 (11.8)	244 (11.7)	
Nationality (*n*, %)			
Swiss	8251 (71.6)	1801 (86.3)	<0.001
Non-Swiss	3269 (28.4)	285 (13.7)	
Major areas (*n*, %)			
Lake Geneva (French)	2387 (20.7)	405 (19.4)	0.029
Midlands (French & German)	2060 (17.9)	387 (18.6)	
Northwest (German)	1677 (14.6)	304 (14.6)	
Zurich (German)	1497 (13.0)	304 (14.6)	
East (German)	1333 (11.6)	251 (12.0)	
Center (German)	1114 (9.7)	219 (10.5)	
South (Italian)	1452 (12.6)	216 (10.4)	

^1^ Differences between the two groups (participants vs. non-participants) were assessed using chi-square tests, respectively two-sample *t* test for age.

**Table 2:** Description of participants, by sex and linguistic region, Swiss Nutrition Survey, 2014–2015.

Characteristics	All	Men	Women	German-Speaking Region ^4^	French-Speaking Region ^4^	Italian-Speaking Region ^4^	*p*-Value ^5^
Participants with 2 complete 24HDR (*n* (%))	2057 (100)	933 (45)	1124 (55)	1341 (65)	502 (24)	214 (10)	
People for weighted analyses (*n* (%))	4,627,878 (100)	2,305,141 (50)	2,322,737 (50)	3,203,780 (69)	1,167,173 (25)	256,925 (6)	
Age ^1^							
(year, mean ± SD)	46.1 ± 15.4	46.9 ± 15.4	45.3 ± 15.3	46.5 ± 15.6	45.0 ± 14.6	46.0 ± 15.7	0.134
Age groups ^1^ (%)							
18–34 years old	28.5	27.4	29.6	27.5	30.7	30.2	0.007
35–49 years old	30.6	29.4	31.8	30.7	31.1	27.3	
50–64 years old	26.9	28.0	25.8	26.5	27.5	28.4	
65–75 years old	14.0	15.2	12.9	15.3	10.7	14.1	
Education: Highest degree ^1^ (%)							
Only primary school or no degree	4.7	4.8	4.6	3.6	6.4	10.3	<0.001
Secondary (e.g., apprenticeship)	42.6	39.2	46.0	41.4	44.5	49.6	
Tertiary (e.g., high technical school, university)	52.7	56.0	49.4	55.0	49.1	40.1	
Self-rep. physical activity ^1^ (%)					8		
Low	10.9	13.0	8.8	11.7	.1	12.8	0.313
Moderate	24.9	23.2	26.7	24.8	25.0	25.8	
High	40.4	43.2	37.6	40.4	41.5	34.8	
Does not know	23.8	20.6	26.9	23.0	25.4	26.5	
Body Mass Index (BMI) ^1^							
(kg/m^2^, mean ± SD)	25.0 ± 4.4	25.9 ± 3.9	24.0 ± 4.7	25.0 ± 4.4	24.8 ± 4.3	25.5 ± 4.9	0.325
BMI categories ^1^ (%)							
Underweight (BMI < 18.5 kg/m^2^)	2.4	0.9	3.8	2.5	2.1	2.1	0.948
Normal (18.5 ≤ BMI < 25 kg/m^2^)	54.2	43.3	65.0	53.8	55.7	52.7	
Overweight (25 ≤ BMI < 30 kg/m^2^)	30.5	41.2	19.8	30.8	29.9	29.9	
Obese (BMI ≥ 30 kg/m^2^)	12.9	14.5	11.3	12.9	12.4	15.4	
Risk of metab. complications ^1^ (%)							
Waist circumference: ≥94 cm (♂); ≥80 cm (♀)	33.1	35.1	31.2	34.4	28.7	37.4	0.028
Waist circumference: ≥102 cm (♂); ≥88 cm (♀)	16.5	16.7	16.3	17.2	14.2	18.8	0.208
Energy Intake: Basal Metabolic Rate (EI:BMR) ^2^							
(mean ± SD)	1.43 ± 0.44	1.44 ± 0.47	1.42 ± 0.41	1.46 ± 0.44	1.39 ± 0.43	1.33 ± 0.50	<0.001
Energy misreporting ^2,3^ (%)							
Under-reporters	16.9	18.9	15.0	15.9	17.6	27.3	0.051
Plausible reporters	81.6	79.3	83.8	82.7	81.1	70.3	
Over-reporters	1.5	1.7	1.2	1.4	1.3	2.4	

^1^ Means and proportions are weighted for sex, age, marital status, major area, household size, and nationality; ^2^ Energy intake is the mean of both 24HDR. BMR was estimated by Schofield equations. Means and proportions are weighted for sex, age, marital status, major area, household size, nationality, season and weekday of 24HDR; ^3^ In under-reporters 23% reported being dieting during one or both 24HDR, respectively 11% in plausible reporters and 4% in over-reporters; ^4^ German-speaking region included the cantons of Aargau, Basel-Land, Basel-Stadt, Bern, Lucerne, St. Gallen, Zurich; French-speaking region: Geneva, Jura, Neuchatel, Vaud, and Italian-speaking region: Ticino; ^5^ Differences between the three linguistic regions were assessed using weighted chi-square tests, respectively ANOVA tests.

**Table 3:** Food subgroup and macronutrient intake, total, and by linguistic region

Swiss Food Pyramid Stage		Daily food Subgroup or Nutrient Intakes, (*g or Kcal for Energy*)	Total Population		Total Population					German	French	Italian	Diff. (*p*-Value) ^3^		
			2 Recalls		Usual Intakes ^2^					Usual Intakes ^2^					
			Crude Mean	Weighted Mean ^1^	Weighted Mean ^1^	SEM ^1^	Weighted P25 ^1^	Weighted P50 ^1^	Weighted P75 ^1^	Weighted Mean ^1^	Weighted Mean ^1^	Weighted Mean ^1^	Ger. vs. Fre.	Ger. vs. Ita.	Fre. vs. Ita.
1	Non-caloric beverages	Water	1199.9	1188.0	1190.6	16.6	715.2	1088.2	1612.1	1215.1	1075.3	1291.6	0.001 *	0.250	0.002
		Tea	295.2	289.6	278.0	10.5	29.3	190.7	432.9	281.6	299.8	162.7	0.457	<0.001 *	<0.001 *
		Coffee	250.5	254.1	246.5	5.8	108.3	220.3	350.0	273.4	214.4	135.4	<0.001 *	<0.001 *	<0.001 *
2	Fruit & vegetables	Vegetables	204.3	203.2	203.1	3.2	137.9	192.3	256.8	205.9	214.4	168.0	0.269	<0.001 *	<0.001 *
		Fruit	171.2	171.0	163.2	4.2	86.4	145.7	220.4	162.0	160.3	140.6	0.855	0.109	0.174
		100% juices	59.9	58.6	60.1	3.0	9.7	34.1	83.2	59.5	62.7	38.0	0.636	0.006	0.006
3	Cereal products & potatoes	Tuber products	49.6	50.7	44.8	1.8	33.8	43.4	61.6	50.9	46.9	49.7	0.470	0.863	0.721
		Bread products	113.7	114.7	111.5	2.4	72.9	102.4	140.0	118.0	101.9	95.6	0.002	0.008	0.477
		Pasta, rice	91.5	93.4	93.3	2.5	55.0	85.0	122.5	88.6	96.8	114.5	0.159	0.019	0.131
		Other cereal products	38.1	38.5	39.3	1.5	18.6	31.8	51.5	37.1	41.5	41.0	0.232	0.443	0.927
4	Protein-based products	Milk	114.1	113.4	110.6	3.9	29.1	73.5	149.9	126.6	71.5	108.9	<0.001 *	0.124	0.001 *
		Yogurt, fresh cheese	61.5	59.8	60.1	2.1	14.9	44.6	89.9	61.7	57.7	35.4	0.411	<0.001 *	0.001 *
		Soft cheese	16.5	16.2	16.1	0.7	7.4	13.4	21.9	15.7	15.2	24.7	0.740	0.002	0.002
		Hard cheese	25.7	27.0	25.6	1.1	12.1	20.8	33.6	26.8	22.8	20.2	0.101	0.029	0.445
		Red meat	36.5	37.2	36.1	1.4	20.4	32.7	46.7	31.7	44.5	42.7	<0.001 *	0.085	0.787
		Other unprocessed meat	26.3	29.0	27.5	5.5	18.7	26.5	34.8	26.1	34.4	33.4	0.417	0.576	0.939
		Processed meat	42.0	42.7	42.3	1.4	20.4	36.1	54.6	43.4	36.7	43.0	0.030	0.938	0.206
		Fish, seafood	19.5	21.0	20.6	1.1	7.9	16.3	28.3	17.7	29.0	34.0	<0.001 *	<0.001 *	0.318
		Other protein-based products	23.6	23.9	22.3	1.2	7.9	15.3	28.5	23.7	20.7	17.5	0.293	0.126	0.453
5	Added fats & nuts	Vegetable oil	12.8	12.4	12.3	0.3	7.7	11.1	15.6	11.4	13.9	15.0	0.003	0.010	0.468
		Butter	8.1	7.9	7.6	0.3	2.7	5.8	10.5	8.1	6.4	3.7	0.004	<0.001 *	<0.001 *
		Cream, fatty sauces, oth. fats	30.9	31.0	30.7	1.07	18.1	27.2	39.4	35.5	22.2	16.7	<0.001 *	<0.001 *	0.045
		Nuts, seeds, olives	9.3	10.5	9.73	0.6	2.2	5.72	12.5	10.7	9.0	5.6	0.197	0.002	0.045
6	Sweets, salty snacks & alcohol	Added sweeteners	22.1	22.1	21.6	0.7	8.9	17.5	29.6	20.7	25.1	17.5	0.010	0.131	0.002
		Cakes, desserts, ice-cream	38.3	39.2	38.7	1.5	19.8	34.3	52.0	38.8	39.0	36.2	0.959	0.601	0.615
		Chocolate products	10.4	10.7	10.1	0.4	3.3	7.5	14.1	10.7	10.1	6.0	0.566	<0.001 *	0.002
		Other sweet products	11.8	11.2	11.1	0.5	2.9	7.5	15.3	11.0	10.2	14.9	0.496	0.088	0.048
		Salty snacks	9.1	9.7	9.15	0.7	2.7	6.1	12.1	8.9	11.1	5.6	0.304	0.064	0.019
		Soft drinks	219.8	240.6	212.2	8.9	41.5	135.7	302.5	245.0	155.3	140.0	<0.001 *	<0.001 *	0.574
		Beer	96.7	107.3	101.5	6.8	7.1	42.5	136.7	109.5	82.2	100.3	0.072	0.651	0.415
		Wine, other alcohols	87.6	91.9	89.5	3.7	20.2	61.3	129.3	81.6	104.5	84.9	0.013	0.757	0.124
Macronutrients		Total energy	2183.1	2225.7	2185.5	16.6	1781.8	2126.4	2526.4	2240.7	2114.3	2025.6	0.001 *	<0.001 *	0.155
		Total proteins	82.6	84.6	82.7	0.7	66.5	80.3	96.4	82.5	83.5	82.0	0.570	0.853	0.614
		Total carbohydrates	230.0	233.0	230.4	2.1	177.9	221.9	274.0	237.3	219.9	215.2	<0.001 *	0.003	0.569
		Total fat	89.0	90.8	89.7	1.2	71.6	87.2	105.2	92.6	89.3	77.7	0.349	<0.001 *	0.006
		Total alcohol	12.8	13.8	13.4	0.5	3.7	8.5	17.7	13.1	14.4	12.0	0.274	0.488	0.170

^1^ Survey weights corrected for non-response based on six socio-demographic parameters (i.e., age, sex, marital status, major area, nationality and household size) and uneven distribution of 24HDR over seasons and weekdays; ^2^ Usual intakes were modelled with the Statistical Program to Assess Dietary Exposure (SPADE) using weighted bootstrap samples and age as covariate; ^3^ Differences between linguistic regions were assessed with Wald tests on the standard errors of the means from weighted bootstrap samples (* *p ≤* 0.001).

**Table 4:** Proportion of participants following the seven food-based dietary guidelines, total, by sex and linguistic region.

Food-Based Dietary Guidelines (FBDG) ^1^	Total Population		Men	Women	German	French	Italian	Diff. (*p*-Value) ^4^		
	2 Recalls		2 Recalls		2 Recalls					
	Crude	Weighted	Weighted	Weighted	Weighted	Weighted	Weighted	Ger.vs. Fre.	Ger. vs. Ita.	Fre. vs. Ita.
	%	% ^3^	% ^3^	% ^3^	% ^3^	% ^3^	% ^3^			
Non-caloric beverages *≥1 L/day of water*, *tea and coffee*	82.8	81.9	77.6	86.2	82.6	80.4	79.8	0.359	0.340	0.864
Fruit and vegetables *≥5 portions/day: max. 1 portion can be provided by 2 dL of 100% fruit or vegetable juices*	18.2	18.1	16.5	19.6	18.1	18.3	17.2	0.920	0.824	0.790
Vegetables *≥3 portions/day: 1 portion* =*120 g*, *30 g if dried*, *2.5 dL of soup*, *and 100 g of sauce*	9.4	9.4	8.8	10.0	8.9	10.1	12.1	0.527	0.291	0.545
Fruit *≥2 portions/day: 1 portion = 120 g, 30 g if dried*	29.3	28.1	24.4	31.8	29.0	27.2	20.0	0.510	0.013	0.063
Dairy products *≥3 portions/day: 1 portion* = *200 mL of milk*, *175 g of yogurt or fresh cheese*, *60 g of soft cheese and 30 g of hard cheese*	21.1	21.7	24.7	18.7	23.4	17.6	19.1	0.036	0.226	0.696
*Total meat* *≤35 g/day of prepared meat*^2^	22.6	22.1	16.1	28.1	23.4	19.3	19.7	0.098	0.340	0.904
Red meat *≤35 g/day of prepared meat*	62.6	62.9	56.0	69.8	64.8	57.7	63.3	0.021	0.703	0.238
Processed meat *≤15 g/day*	43.0	41.7	31.8	51.6	40.0	46.0	43.1	0.054	0.477	0.542
Vegetable oil *≥25 g/day*	13.8	13.5	15.2	11.7	11.8	15.4	24.5	0.077	<0.001*	0.020
Nuts, seeds, and olives *≥25 g/day*	6.2	6.2	5.9	6.6	6.7	5.9	2.4	0.634	0.110	0.174
Alcohol *≤30 g (♂) or* *≤15 g (♀*) of pure alcohol	77.9	76.7	75.7	77.7	78.2	72.2	79.0	0.029	0.814	0.094
All 7 FBDG above	0.1	0.1	0.0	0.2	0.1	0.0	0.0	*Not applicable*		
At least 3 FBDG	41.9	40.5	37.3	43.7	42.4	36.0	38.1	0.037	0.341	0.666

^1^ Reference from the Swiss Food Pyramid; ^2^ Thirty-five grams of cooked/prepared meat per day corresponds to the guideline of 2–3 portions of 110 g of any type of raw meat per week (max.1 portion of processed meat per week); ^3^ Survey weights corrected for non-response based on six socio-demographic parameters (i.e., age, sex, marital status, major area, nationality and household size) and uneven distribution of 24HDR over seasons and weekdays; ^4^ Differences between linguistic regions were assessed using Wald tests on survey-weighted logistic regression coefficients (* *p ≤* 0.001).

## Supplementary Materials

The following are available online at www.mdpi.com/2072-6643/9/11/1163/s1, Table S1: Description of foods and beverages included in each food group by food pyramid stage, Table S2: Food group and macronutrient intake, by sex, Table S3: Food group and macronutrient intake, by age, Figure S1: Distribution of 24HDR over weekdays and seasons, Figure S2: Participation classification, Figure S3: Heatmap from Switzerland presenting the survey participants’ geographical provenance.

## Abbreviations

The following abbreviations are used in this manuscript:

24HDR	24 h dietary recalls
GD	GloboDiet^®^
FBDG	Food-based dietary guidelines
EI:BMR	Ratio of reported Energy Intake and Basal Metabolic Rate
EPIC study	European Prospective Investigation into Cancer and Nutrition study
IARC	International Agency for Research on Cancer
ICMJE	International Committee of Medical Journal Editors
IPAQ	International Physical Activity Questionnaire
ISRCTN	International Standard Randomised Controlled Trial Number
MSM	Multiple Source Method
PAL	Physical activity level
SPADE	Statistical Program to Assess Dietary Exposure
STROBE	STrengthening the Reporting of OBservational studies in Epidemiology
WHO	World Health Organization
