# Supplementary material for: Major Differences in Diet across Three Linguistic Regions of Switzerland: Results from the First National Nutrition Survey menuCH
Source: Nutrients. 2017 Oct 25;9(11):1163. doi: 10.3390/nu9111163 (PMC5707635; doi:10.3390/nu9111163)
Supplement: Supplementary file 1 [file nutrients-09-01163-s001.pdf]

Online Supplementary Material

Major differences in diet across three linguistic regions of Switzerland:

results from the first national nutrition survey *menuCH*

Table of content

- *Table S1.* *Description of foods and beverages included in each food group by food pyramid stage* *2*
- *Table S2.* *Food group and macronutrient intake, by sex* *4*
- *Table S3.* *Food group and macronutrient intake, by age* *5*
- *Figure S1.* *Distribution of 24 HDR over weekdays and seasons* *6*
- *Figure S2.* *Participation classification* *7*
- *Figure S3.* *Heatmap from Switzerland presenting the survey participants' geographical provenance* *8*

Table S1. Description of foods and beverages included in each food group by Swiss Food Pyramid stage

Swiss Food Pyramid stage		Food subgroup name	Foods included in this group
Non-caloric beverages	1	Water	All types of water (e.g. tap, mineral, still, carbonated), *consumed 99 of times without added sugars/artificial sweeteners.*
	2	Tea	Tea, herbal and fruit tea, *consumed 98 of times without added sugars/artificial sweeteners.*
	3	Coffee	Coffee (e.g. instant, from capsules), and coffee with milk or cream (e.g. cappuccino), coffee substitutes (e.g. chicory), *consumed 98 of times without added sugars/artificial sweeteners.*
Fruit & vegetables	4	Vegetables	All types of vegetables, green leaves, sprouts, mushrooms, seaweeds, sweet corn, snow peas, fresh green beans, and onions: raw, cooked, dried, canned, in puree, pickled, in soups, in sauce (e.g. tomato sauce), on pizza and quiches, and in sandwiches.<br>Except: avocadoes, olives, herbs, vegetable juices, nor if contained in small amounts in salty snacks, bread, nor sauces.
	5	Fruit	All types of fruit: raw, cooked, dried, in puree/compote, and in pies made essentially with fruit (e.g. apples in apple pies).<br>Except: fruit juices, fruit jams, candied fruit, nor if contained in small amounts in yogurts, cakes, ice-cream nor other sweets.
	6	100% juices	100% fruit and vegetable juices, and smoothies, *without added sugars.*
Cereal products & potatoes	7	Tuber products	All types of unprocessed tubers (e.g. potatoes, sweet potatoes) and potato products (e.g. rösti, gnocchi, French fries, mashed potatoes).
	8	Bread products	All types of bread (e.g. baguette, bread rolls, milk bread), flat bread, croissants, and crisp bread (e.g. rice crackers, Swedish rolls).<br>Except: stuffed croissants and sweet pastries (e.g. chocolate croissants).
	9	Pasta and rice	Plain pasta (e.g. penne, spaghetti), stuffed pasta (e.g. ravioli, tortellini), schupfnudeln, spatzli, rice and rice noodles.
	10	Other cereal products	Natural cereal flakes and bran, oatmeal, natural birchermuesli mixes, dried wheat germs, *all without added sugars/artificial sweeteners and without major food processing*, legumes, wheat and other cereal grains (e.g. barley, quinoa), cereal semolina, flours and starches, and all types of dough.
Protein-based products	11	Milk	Mammals' milk, branded fermented milk drinks, yogurt drink and buttermilk, *in liquid form, consumed 96 of times without added sugars/artificial sweeteners.*
	12	Yogurt and fresh cheese	Mammals' yogurt, branded fermented milk and kefir, fresh cheese (e.g. quark, cottage cheese, ricotta), *in semi-solid form, consumed 53 of times with added sugars/artificial sweeteners.*
	13	Soft cheese	Mammals' spread cheese, processed/melted cheese, and soft cheese.
	14	Hard cheese	Mammals' hard cheese.
	15	Red meat	Fresh meat and offal from beef, veal, pork, lamb, mutton, horse, goat, rabbit, and wild red meat (e.g. venison/deer).

	16	Other unprocessed meat	Fresh meat and offal from chicken, turkey, duck, goose, ostrich, frog, and unprocessed meat and offal from any unspecified animals.
	17	Processed meat	Sausages, cold cuts, smoked and cured meat (e.g. ham, bacon, salami, corned beef, beef jerky), meat-based sauces (e.g. Bolognese sauce), and meat-based spread (e.g. liver spread).
	18	Fish and seafood	Fresh fish, seafood and snails, and processed fish and seafood products (e.g. fish in crumb, surimi).
	19	Other protein-based products	Eggs, milk and yogurt substitutes (e.g. soya milk), tofu, quorn, and other vegetarian products (e.g. vegetarian sausages), dietetic products rich in proteins (e.g. meal replacements, protein shakes).<br>Except: eggs contained in small amounts in cakes nor salty snacks, nor sauces.
Added fats & nuts	20	Vegetable oil	Added vegetable oils (e.g. rapeseed oil, olive oil, sunflower oil) or in dressing sauces (e.g. French dressing, Italian dressing, estimated percentage of vegetable oils in dressings: 40).
	21	Butter	Added butter to cook and spread on bread.
	22	Cream, fatty sauces and other fats	Mammals' cream, margarine, coco fat, added animal fats (e.g. pig fat), any unspecified added fats, mascarpone, sauces rich in oil or butter (e.g. mayonnaise, pesto, sauce café de Paris), other sauces rich in cream or other fats (e.g. carbonara, cocktail sauce, hummus, satay sauce), and coco milk.
	23	Nuts, seeds, and olives	Dried nuts and seeds (e.g. almonds, coconut, pumpkin seeds), olives, avocadoes, guacamole, *all with or without salt.*
Sweets, salty snacks & alcohol	24	Added sweeteners	Beet and cane sugar, jams, jelly, honey, syrups, polyols, sweet sauces (e.g. caramel and chocolate sauce, maple syrup), sweet spreads (e.g. chocolate spread, peanut butter), sweet topping (e.g. icing), and chocolate powder.<br>Except: stevia and artificial sweeteners.
	25	Cakes, desserts, ice-cream	Ice-cream, sorbet, iced cakes, desserts made with dairy products (e.g. pudding, chocolate mousse, tiramisu), sweet cakes, pies and tarts (e.g. brownies, lemon cakes, muffins), and sweet pastries (e.g. chocolate croissants, doughnuts, waffles).
	26	Chocolate products	Pure chocolate, chocolate products and confectionary made with chocolate (e.g. filled chocolate, pralines, branded chocolate bars).
	27	Other sweet products	Sweet and dried biscuits (e.g. shortbread, amaretto, chocolate chip cookies), branded breakfast cereals (e.g. corn-flakes) and industrial birchermuesli mixes, *processed and with added sugars/artificial sweeteners,* sweets and confectionery (e.g. marzipan, candied fruit, lollipop, marshmallows, nougat), sweet and sports cereal bars (e.g. energy bars).
	28	Salty snacks	Crisps, salty popcorn, dried salty crackers (e.g. golden fish biscuits, sticks, pretzels), cocktail canapés, crostini/bruschetta, spring rolls, ham croissants, and sausage rolls.
	29	Soft drinks	Sweetened and sugar-free soft drinks, sports and energy drinks, fizzy drinks, ice tea, diluted syrup, drinks made with fruit juices (e.g. lemonades, nectars), and alcoholic drink substitutes (e.g. alcohol-free beers), 'schorle' (i.e. juices mixed with water), *consumed 18 of times with artificial sweeteners only.*
	30	Beer	Beers (with alcohol), ciders and shandy.
	31	Wine and other alcoholic beverages	White and red wine, champagne, wine products (e.g. sangria, punch/bowle with alcohol), port, vermouths, liquors, spirits, cocktails and long drinks, 'alcopops', and any other alcoholic drinks (e.g. coffee with spirit).

Online Supplementary Material

Table S2. Food subgroup and macronutrient intake, by sex

Daily food subgroup or nutrient intakes, *(g or kcal for energy)*	Men				Women			
	Usual intakes[1]				Usual intakes[1]			
	Weighted mean	Weighted P25	Weighted P50	Weighted P75	Weighted mean	Weighted P25	Weighted P50	Weighted P75
Water	1218.2	672.9	1099.4	1632.6	1221.5	755.2	1136.4	1593.9
Tea	194.6	2.3	68.3	314.1	362.8	115.6	301.1	526.5
Coffee	267.0	128.8	237.6	369.3	228.1	90.3	203.9	330.3
Vegetables	197.2	133.2	184.6	247.8	209.5	141.4	196.5	263.9
Fruit	147.4	76.6	128.7	198.5	173.7	97.2	157.8	232.4
100% juices	59.7	12.3	37.0	82.3	57.2	7.9	32.4	81.5
Tuber products	54.8	33.7	51.8	72.8	44.8	35.3	43.4	52.8
Bread products	137.5	95.6	129.1	170.3	90.4	61.9	84.6	112.6
Pasta and rice	115.1	72.2	106.6	148.7	72.6	42.7	67.5	96.7
Other cereal products	39.5	18.4	31.7	51.9	37.5	18.3	30.7	49.0
Milk	120.1	27.4	74.1	161.0	105.7	33.9	78.0	146.3
Yogurt and fresh cheese	58.5	9.0	38.5	91.2	61.4	21.2	48.8	88.9
Soft cheese	19.3	7.6	15.7	27.1	13.8	7.4	11.9	18.1
Hard cheese	29.6	13.5	23.3	38.5	20.8	10.8	17.8	27.5
Red meat	47.6	28.1	42.5	61.4	25.9	15.3	23.2	33.5
Other unprocessed meat	33.8	17.0	29.0	45.5	26.0	22.9	25.7	28.9
Processed meat	55.5	30.4	48.5	73.1	29.6	14.4	24.5	39.2
Fish and seafood	21.7	6.5	15.8	30.9	19.6	9.3	16.1	26.1
Other protein-based products	23.4	8.5	16.1	29.7	22.1	7.5	14.9	28.2
Vegetable oil	12.6	7.0	10.8	16.1	12.1	8.5	11.4	14.9
Butter	8.5	2.9	6.7	12.1	6.6	2.6	5.1	9.0
Cream, fatty sauces and other fats	35.1	21.4	31.2	44.5	26.0	15.0	23.0	33.6
Nuts, seeds, olives	9.1	1.9	4.7	10.9	11.3	2.8	7.1	14.7
Added sweeteners	26.6	10.4	21.4	36.8	18.7	8.5	15.7	25.6
Cakes, desserts, ice-cream	43.2	20.4	38.3	60.5	34.5	19.5	30.5	45.0
Chocolate products	10.1	2.3	6.7	14.3	10.5	4.5	8.4	14.2
Other sweet products	12.2	2.0	7.1	17.1	11.0	3.8	8.0	14.8
Salty snacks	10.8	2.6	6.8	14.4	7.8	3.5	6.3	10.4
Soft drinks	290.3	60.7	206.5	430.3	154.9	33.0	94.4	212.8
Beer	185.8	31.4	109.2	259.4	31.8	3.4	15.3	42.9
Wine and other alcoholic beverages	106.1	24.2	73.7	154.2	71.5	17.4	49.7	103.5
Total energy	2537.8	2104.6	2487.9	2914.1	1899.2	1624.4	1871.7	2145.2
Total proteins	98.3	80.5	96.4	114.0	70.1	59.4	68.6	79.3
Total carbohydrates	264.8	206.8	256.5	313.6	202.2	162.9	197.5	236.3
Total fat	101.9	81.3	99.3	119.6	79.2	65.6	77.6	91.2
Total alcohol	18.4	6.9	13.7	24.8	8.7	2.0	5.0	11.1

[1] Usual intakes were modelled with SPADE using age as covariate. Survey weights corrected for non-response based 6 socio-demographic parameters (i.e., age, sex, marital status, major area, nationality and household size) and uneven distribution of 24HDR over seasons and weekdays.

Online Supplementary Material

Table S3. Food subgroup and macronutrient intake, by age

Daily food subgroup or nutrient intakes, *(g or kcal for energy)*	18-34 years old		35-49 years old		50-64 years old		65-75 years old	
	Usual intakes[1]		Usual intakes[1]		Usual intakes[1]		Usual intakes[1]	
	Weighted mean	Weighted P50	Weighted mean	Weighted P50	Weighted mean	Weighted P50	Weighted mean	Weighted P50
Water	1471.4	1382.4	1267.5	1176.2	1083.7	990.8	923.1	832.5
Tea	223.2	120.6	264.6	174.4	298.3	219.8	361.7	301.4
Coffee	152.0	116.5	263.5	238.2	303.2	275.4	276.9	249.7
Vegetables	185.2	172.2	207.1	194.5	213.5	200.3	216.7	204.1
Fruit	127.3	110.4	151.7	135.4	181.3	164.8	214.9	199.2
100% juices	62.7	34.4	60.5	33.7	58.2	33.8	58.1	35.0
Tuber products	49.7	47.6	47.0	44.6	48.2	45.7	53.4	50.9
Bread products	115.2	105.7	112.9	104.0	109.2	100.4	106.6	98.0
Pasta and rice	111.3	103.4	99.6	92.0	81.2	73.5	71.8	64.6
Other cereal products	46.3	38.4	41.1	33.8	36.0	29.2	29.3	23.5
Milk	127.7	86.8	115.8	79.0	100.0	65.9	89.7	59.1
Yogurt and fresh cheese	52.1	35.7	54.4	38.2	66.2	52.0	73.7	60.9
Soft cheese	15.6	12.9	17.0	14.2	16.0	13.2	15.6	13.1
Hard cheese	20.5	16.4	25.8	21.2	27.2	22.3	31.3	26.1
Red meat	33.3	29.1	38.2	33.8	36.1	31.9	35.9	31.6
Other unprocessed meat	37.0	35.2	29.4	28.0	23.3	22.0	17.0	15.9
Processed meat	46.2	39.2	41.4	35.0	38.2	32.1	36.8	30.8
Fish and seafood	18.0	13.5	22.8	18.1	22.7	17.9	17.7	13.0
Other protein-based products	25.3	17.6	22.8	15.8	20.8	14.3	18.8	12.7
Vegetable oil	11.5	10.3	12.6	11.4	12.7	11.4	12.4	11.1
Butter	5.4	3.8	6.9	5.4	8.2	6.6	11.4	9.7
Cream, fatty sauces and other fats	31.7	28.0	31.8	28.1	30.5	27.0	29.5	26.1
Nuts, seeds, olives	10.0	5.8	10.1	5.9	9.6	5.7	8.9	5.2
Added sweeteners	18.0	14.1	21.4	17.4	22.2	18.2	26.8	22.7
Cakes, desserts, ice-cream	36.6	31.4	41.4	36.6	38.0	33.3	37.1	32.8
Chocolate products	12.3	9.5	11.3	8.7	8.4	6.2	7.0	4.9
Other sweet products	11.0	7.2	11.2	7.5	10.8	7.3	11.4	8.1
Salty snacks	12.9	9.5	10.1	7.2	6.0	3.7	6.4	4.3
Soft drinks	317.4	244.0	219.7	149.9	161.7	100.1	106.0	51.3
Beer	137.2	68.6	118.0	59.8	80.5	33.3	45.7	10.5
Wine and other alcoholic beverages	55.9	27.8	81.2	54.0	107.0	81.1	132.5	107.6
Total energy	2254.6	2195.9	2222.4	2164.4	2153.7	2096.1	2052.8	1999.3
Total proteins	85.2	82.7	85.0	82.5	81.6	79.2	76.2	74.0
Total carbohydrates	251.5	243.3	234.9	226.8	219.4	211.5	205.3	198.0
Total fat	90.8	88.3	91.2	88.7	88.7	86.2	86.7	84.4
Total alcohol	9.4	5.7	12.5	8.2	15.3	10.3	18.7	12.9

[1] Usual intakes were modelled with SPADE. Survey weights corrected for non-response based 6 socio-demographic parameters (i.e., age, sex, marital status, major area, nationality and household size) and uneven distribution of 24HDR over seasons and weekdays.

Online Supplementary Material

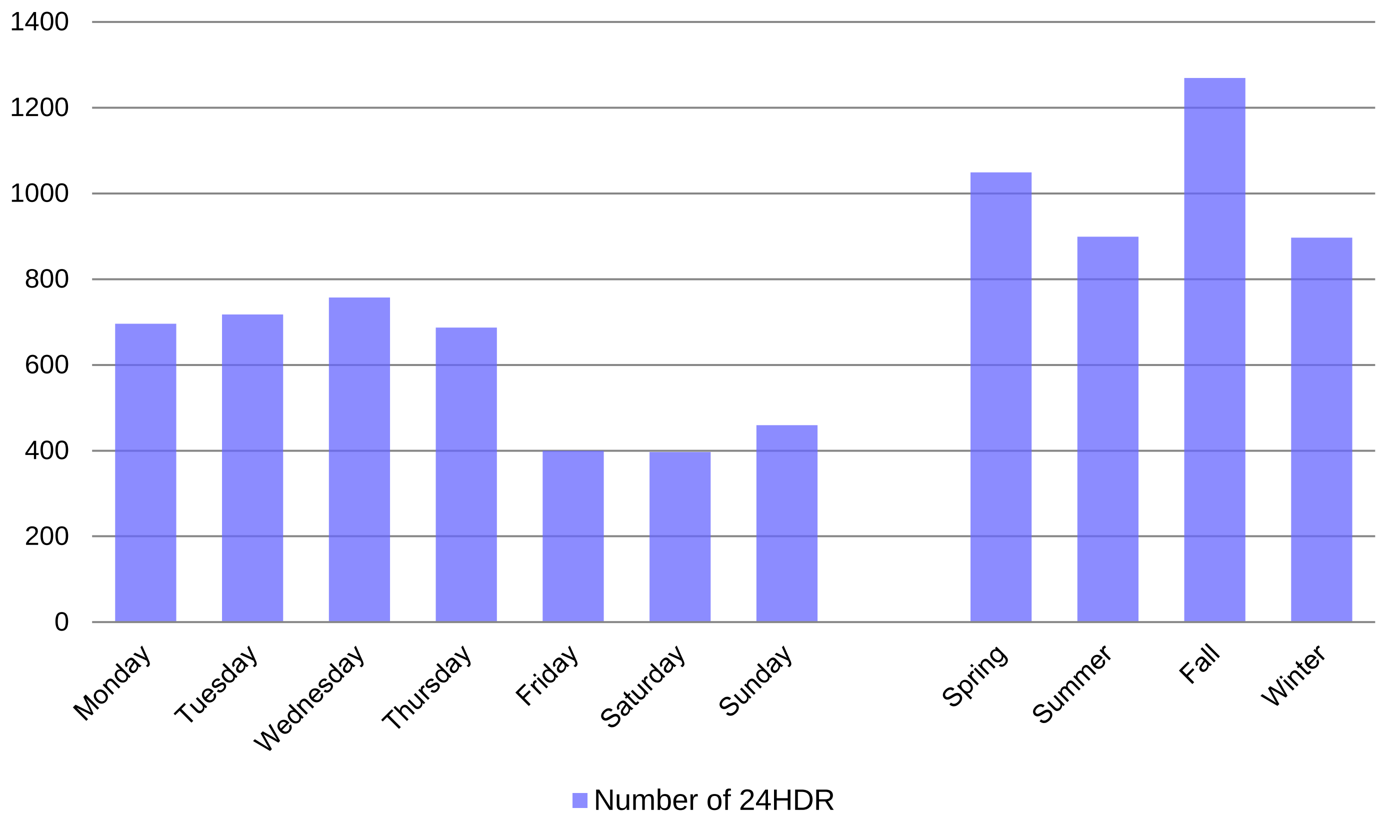

Figure S1. *Distribution of 24 HDR days (n=4,114) over weekdays and seasons*

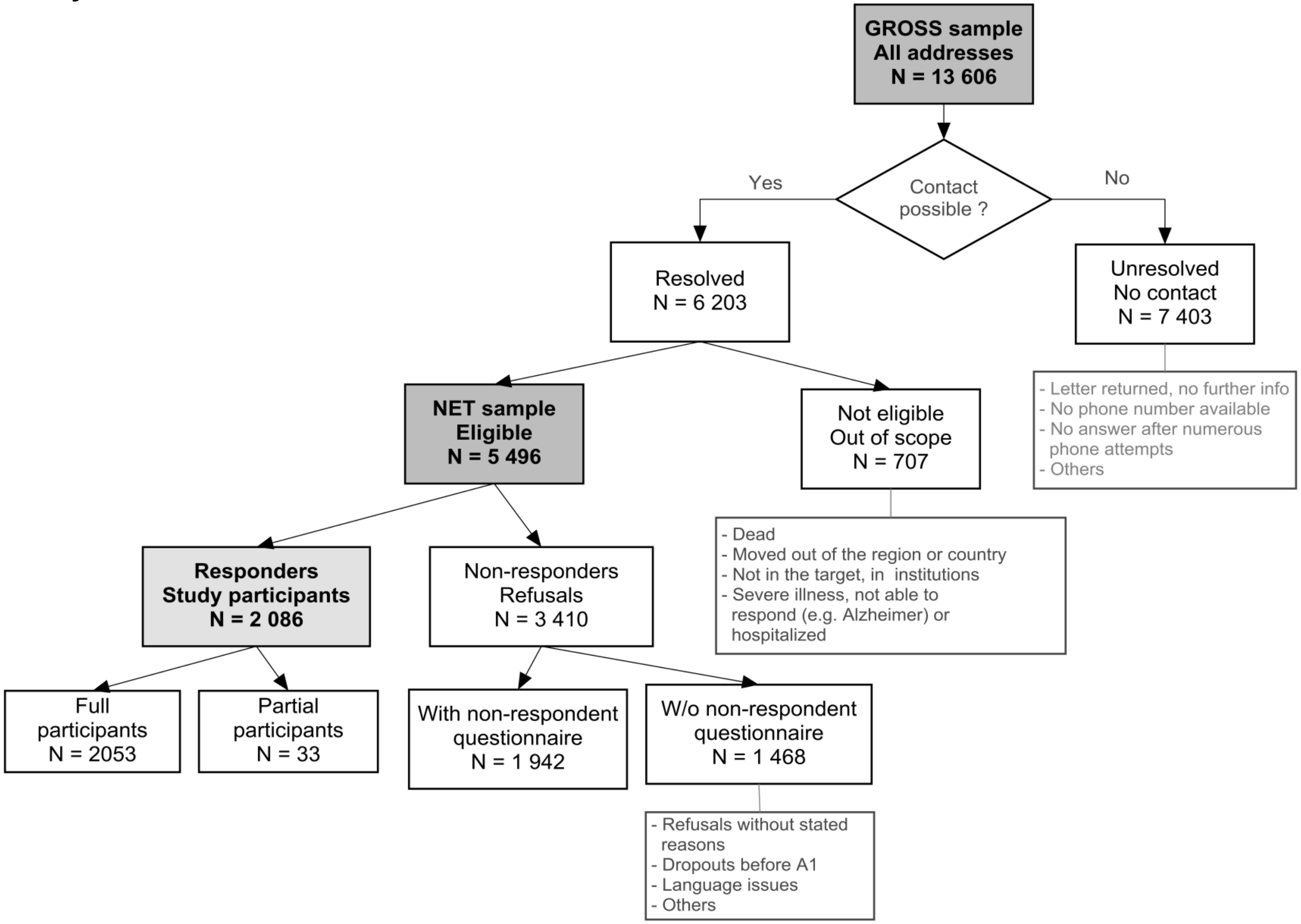

Figure S2. *Participation classification, following 2014 European Food Safety Authority's classification recommendations for food consumption surveys. Full participation implies the entire dataset was available: two 24HDR, a complete questionnaire, measured (or self-reported when impossible) weight and height.*

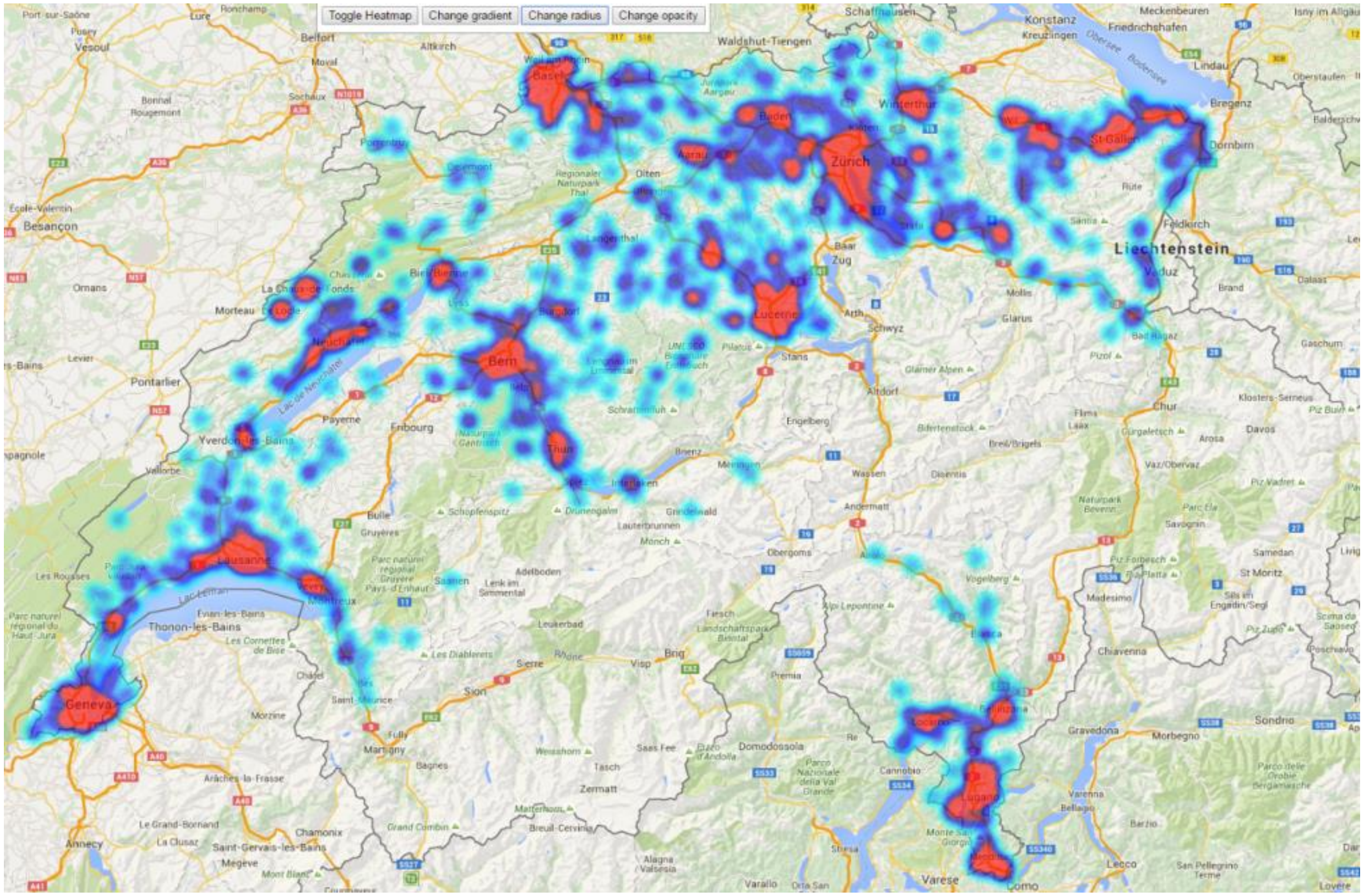

Figure S3. *Heatmap from Switzerland presenting the survey participants' geographical provenance based on the addresses provided by the Federal Statistical Office. Geolocalization through Google Maps.*
